# Imported Malaria in China, 2012

**DOI:** 10.3201/eid2010.140595

**Published:** 2014-10

**Authors:** Jun Feng, He Yan, Xin-yu Feng, Li Zhang, Mei Li, Zhi-gui Xia, Ning Xiao

**Keywords:** malaria, imported malaria, parasites, Plasmodium species, workers, China

**To the Editor:** Imported malaria has become a major public health challenge in China. Despite an 89.8% decrease in total cases from 2008 (26,873) through 2012 (2,729), the proportion of imported malaria cases has increased from 14.7% to 89.0% (*1*). We analyzed the malaria situation in China in 2012 by using data obtained from the national information reporting system of infectious diseases.

In this analysis, an imported case of malaria was defined as case of malaria acquired in a known malarious area outside China. In China, the following criteria for imported malaria must be simultaneously met: 1) the patient was given a diagnosis of malaria; 2) the patient had a travel history to malaria-endemic areas outside China during malaria transmission season; and 3) the onset time for malaria for the person was <1 month after returning to China during the local transmission season. This definition of malaria was based on the latent period for all *Plasmodium* species reported in China.

Of 2,729 malaria cases reported in 2012, a total of 2,428 (89.0%) were imported. Fifteen of these cases were in persons who died of infection with *P. falciparum*. In the imported cases, 4 *Plasmodium* species were identified: *P. falciparum* (n = 1,423 [58.6%]), *P. vivax* (n = 909 [37.5%]), *P. ovale* (n = 42 [1.7%]), *P. malariae* (n = 20 [0.8%]), and mixed infections (n = 21 [0.9%]). Among imported cases, 13 (0.5%) were clinically diagnosed.

Chinese workers who returned from Africa (n = 1,458 [60.0%]) had most cases imported malaria. A total of 37 countries in Africa were sources of imported cases. Most cases were acquired in Ghana (n = 241 [9.9%]), Equatorial Guinea (n = 233 [9.6%]), and Nigeria (n = 197 [8.1%]). Case-patients were predominantly infected by *P. falciparum* (n = 1,187 [81.4%]). Southeast Asia (n = 895 [36.9%]), including Myanmar (n = 764 [31.5%]), Cambodia (n = 49 [2.0%]), and Laos (n = 36 [1.5%]), was another major source of imported cases. These case-patients were infected mostly with *P. vivax* (n = 658 [73.5%]) (Table).

**Table:** Malaria cases imported into China from other countries, by country and *Plasmodium* species, 2012

Country of origin	Total, n = 2,428, no. (%)	Species, no. (%)					
		*P. falciparum*, n = 1,423	*P. vivax*, n = 909	*P. malariae*, n = 20	*P. ovale*, n = 42	Mixed, n = 21	Unclassified, n = 13
Africa	1,458 (60.0)	1,187 (83.4)	192 (21.1)	16 (80.0)	40 (95.2)	12 (57.1)	11 (84.6)
Ghana	241	207	21	4	2	3	4
Equatorial Guinea	233	187	25	2	15	3	1
Nigeria	197	172	19	1	5	0	0
Angola	151	127	13	4	5	0	2
Guinea	60	56	3	0	1	0	0
Sudan	50	34	16	0	0	0	0
Liberia	45	32	10	1	1	1	0
Republic of Congo	43	34	4	1	2	2	0
Sierra Leone	42	36	3	0	1	1	1
Gabon	37	31	4	0	2	0	0
Democratic Republic of Congo	36	28	6	0	1	1	0
Ethiopia	30	9	21	0	0	0	0
Mozambique	20	18	2	0	0	0	0
Cameroon	18	12	6	0	0	0	0
Côte d’Ivoire	17	17	0	0	0	0	0
Mali	17	15	2	0	0	0	0
Tanzania	17	15	2	0	0	0	0
Zambia	16	15	1	0	0	0	0
Uganda	16	11	4	0	1	0	0
South Sudan	16	16	0	0	0	0	0
Chad	11	8	3	0	0	0	0
Malawi	10	8	1	0	1	0	0
Central African Republic	8	7	0	1	0	0	0
South Africa	8	4	2	1	1	0	0
Senegal	5	4	1	0	0	0	0
Benin	5	4	1	0	0	0	0
Burkina Faso	5	5	0	0	0	0	0
Kenya	5	4	1	0	0	0	0
Madagascar	5	5	0	0	0	0	0
Niger	4	4	0	0	0	0	0
Libya	3	1	2	0	0	0	0
Zimbabwe	2	2	0	0	0	0	0
Togo	2	2	0	0	0	0	0
Rwanda	2	1	1	0	0	0	0
Mauritania	1	0	1	0	0	0	0
Egypt	1	1	0	0	0	0	0
Algeria	1	0	1	0	0	0	0
Unknown	78	55	16	1	2	1	3
Southeast Asia	895 (36.9)	224 (15.7)	658 (72.4)	3 (15.0)	1 (2.4)	9 (42.9)	0
Myanmar	764	198	557	2	1	6	0
Cambodia	49	1	46	1	0	1	0
Laos	36	4	32	0	0	0	0
Indonesia	35	17	16	0	0	2	0
East Timor	3	0	3	0	0	0	0
Vietnam	5	3	2	0	0	0	0
Thailand	1	0	1	0	0	0	0
Malaysia	1	1	0	0	0	0	0
Unknown	1	0	1	0	0	0	0
Southern Asia	45 (1.8)	4 (0.3)	39 (4.3)	0	1 (2.4)	0	1 (7.7)
Pakistan	29	4	24	0	1	0	0
India	14	0	13	0	0	0	1
Afghanistan	2	0	2	0	0	0	0
Eastern Asia	2 (0.1)	0	2 (0.2)	0	0	0	0
South Korea	1	0	1	0	0	0	0
North Korea	1	0	1	0	0	0	0
Oceania	6 (0.2)	2 (0.2)	4 (0.5)	0	0	0	0
Papua New Guinea	6	2	4	0	0	0	0
Latin America	1 (0.1)	0	1 (0.1)	0	0	0	0
Brazil	1	0	1	0	0	0	0
Unknown	21 (0.9)	6 (0.4)	13 (1.4)	1 (5.0)	0	0	1 (7.7)

Imported cases increased during April, reached a peak during May (n = 297 [12.2%]), and decreased during July. This trend was caused by workers returning to China to perform agricultural work during this period. The male:female patient ratio was 14.6:1 (n = 2,272 male patients:156 female patients); most (n = 2,240 [95.2%]) mobile laborers are men. The mean age of persons with imported cases was 40.8 years. Most (n = 1,813 [74.7%]) of these persons were 15–44 years of age and few (n = 5 [0.2%]) were <5 years of age.

Persons with imported cases were detected in 29 provinces (Hong Kong, Macao, and Taiwan did not join the information system). Yunnan (n = 690 [28.4%]), Guangxi (n = 209 [8.6%]), and Jiangsu (n = 197 [8.1%]) Provinces had the largest number of imported cases.

Our analysis indicated that imported malaria poses major challenges to the malaria elimination program in China. One challenge is the increasing investment in overseas work and increasing numbers of Chinese persons who are working abroad. The total number of Chinese laborers and travelers abroad in 2012 was estimated to be 0.5 million and 83.2 million persons, respectively; these numbers increased by 24.6% and 44.9%, respectively, from numbers in 2010. Another reason for the increasing proportion of imported malaria cases was a sharp decrease in locally acquired infections. There were only 246 locally acquired cases in 2012, a decrease of 94.2% from the number of locally acquired cases in 2010 (*2*).

Because imported malaria is widely distributed throughout China, the disease could be introduced into malaria-free localities during the transmission season, especially when a large number of cases are clustered in areas in which *Anopheles* species mosquitoes are prevalent. Additional studies are needed to determine the susceptibility of *Anopheles* species mosquitoes in China to *Plasmodium* species that cause human malaria.

In summary, imported malaria poses a severe threat to the malaria elimination program in China (*3*). For effective management of imported malaria, surveillance systems need to be carefully planned and well managed to ensure timely recognition and prompt response. Effective mechanisms of multisectoral coordination and cooperation should be established and strengthened. In addition, health education information on malaria risks and protection should be provided to all mobile laborers and other travelers before their traveling abroad and after returning home. Labor and travel agencies should provide travelers with essential preventive measures. This information should also be provided to entry and exit border stations and to local Centers for Disease Control and Prevention so that timely malaria tracking can be implemented. Training should also be provided to physicians to ensure provision of accurate diagnosis and appropriate treatment. For local health agencies, prompt case verification and response are required to ensure elimination of residual potential reservoirs and prevention of local transmission caused by imported pathogens.

## References

[1] Zhou SS, Wang Y, Fang W, Tang LH. Malaria situation in the People’s Republic of China in 2008 [in Chinese]. Zhongguo Ji Sheng Chong Xue Yu Ji Sheng Chong Bing Za Zhi. 2009;27:455–6. 20232622

[2] Zhou SS, Wang Y, Li Y. Malaria situation in the People’s Republic of China in 2010 [in Chinese]. Zhongguo Ji Sheng Chong Xue Yu Ji Sheng Chong Bing Za Zhi. 2011;29:401–3. 24822335

[3] Liu Y, Michelle SH, Zhou HY, Wang WM, Cao YY, Roly DG, Malaria in overseas labourers returning to China: an analysis of imported malaria in Jiangsu Province, 2001–2011. Malar J. 2014;13:29. 10.1186/1475-2875-13-29 24460982 PMC3922785

